# Assessment of the Type and Degree of Genomic Instability in Gliomas

**DOI:** 10.3390/ijms27062678

**Published:** 2026-03-15

**Authors:** Nejla Ademović, Marina Milić, Tijana Tomić, Blagoje Murganić, Ivan Milić, Nasta Tanić, Nikola Tanić

**Affiliations:** 1 Department of Neurobiology, Institute for Biological Research “Siniša Stanković”, National Institute of Republic of Serbia, University of Belgrade, 11000 Belgrade, Serbia; nejla.ademovic@ibiss.bg.ac.rs; 2 Clinic for Neurosurgery, University Clinical Center of Serbia, Koste Todorovica 6, 11000 Belgrade, Serbia; dr.marina.r@gmail.com (M.M.); drivanmilic@gmail.com (I.M.); 3 Faculty of Medicine, University of Belgrade, 11000 Belgrade, Serbia; 4 Department of Radiobiology and Molecular Genetics, Institute of Nuclear Sciences “Vinča”, National Institute of Republic of Serbia, University of Belgrade, 11000 Belgrade, Serbia; tijana.tomic@vin.bg.ac.rs (T.T.); blagoje@vin.bg.ac.rs (B.M.); nastad@vin.bg.ac.rs (N.T.); 5 Department of Natural Sciences and Mathematics, Field of Biology, State University of Novi Pazar, 36300 Novi Pazar, Serbia

**Keywords:** genomic instability (GI), microsatellite instability (MIN), chromosomal instability (CIN), glioblastoma IDH wild-type, astrocytoma IDH mutant

## Abstract

Glial brain tumours, including astrocytoma IDH (Isocitrate Dehydrogenase) mutant and glioblastoma IDH wild-type, are highly malignant brain tumours with poor clinical outcomes. Genomic instability, encompassing microsatellite (MIN) and chromosomal instability (CIN), drives tumour heterogeneity and evolution. In this study, genomic instability was analysed in 85 patients using AP-PCR (Arbitrarily Primed Polymerase Chain Reaction) by comparing tumour and normal tissue (blood) DNA profiles of the same patient. Both types of alterations were present in all analysed samples, contributing almost equally to the total level of genomic instability. The dominant pattern of genomic instability in our cohort was low overall instability, predominantly manifesting as low-degree microsatellite instability. A general decrease in genomic instability was observed with increasing tumour grade. Glioblastoma IDH wild-type was more prevalent in older patients, whereas astrocytoma IDH mutant predominated in younger individuals. Notably, low genomic instability (both MIN and CIN) was associated with poorer survival in patients over 50 years of age. Females, compared to males, exhibited higher MIN in grade 2 tumours and elevated CIN in grade 4 tumours. Our results confirm that genomic instability contributes to tumour progression, MIN being the pivotal factor, and could serve as a prognostic biomarker in malignant gliomas.

## 1. Introduction

Glial brain tumours are the most malignant and aggressive diffusely infiltrating tumours of the brain. They represent 25.1% of all primary brain tumours and 80.8% of all malignant brain tumours in adults [1]. These tumours are associated with poor prognosis and limited survival. The median survival for patients with glioblastoma is 9.2 months, increasing to 16.1 months after treatment [2]. In an updated version of the classification of brain cancer issued by the World Health Organization (WHO) in 2021, gliomas belong to the group of adult-type diffuse tumours and include two major tumour types, astrocytoma IDH mutant and glioblastoma IDH wild-type, in further text astrocytoma and glioblastoma, which were the focus of our study [3]. Although histologically similar, these two tumour types differ in their genetic and epigenetic profiles, clinical course of disease, presence or absence of precursor lesions, molecular pathways of pathogenesis, therapeutic approaches, and clinical outcomes [4,5,6,7]. Glioblastoma is a highly malignant primary tumour that typically arises de novo [4,5]. These tumours are more frequently observed in older male patients and are associated with rapid progression and poor prognosis [8,9]. They exhibit significant molecular heterogeneity and are characterized by recurrent genetic alterations, including *EGFR* (Epidermal Growth Factor Receptor) amplification, *PTEN* (Phosphatase and TENsin homolog) mutations, loss of heterozygosity on chromosome 10, gain of the entire chromosome 7, and mutations in the *TERT* (Telomerase Reverse Transcriptase) promoter [3,10]. Median survival following diagnosis ranges from 8 to 13 months [1,11]. Astrocytoma is a secondary glial tumour that arises from low-grade progression. They occur in younger patients (mean < 45 years of age) and are more common in the female population [4,5]. These tumours are characterized by recurrent mutations in *IDH1/2*, *TP53* (Tumour Protein 53), and *ATRX* (Alpha-thalassemia intellectual disability, X-linked), as well as methylation of the *MGMT* (O6-Methylguanine-DNA Methyltransferase) promoter [9,12]. According to the 2021 WHO classification of central nervous system (CNS) tumours, astrocytoma is categorized into CNS WHO grades 2, 3, and 4 [3]. The median overall survival for patients with these tumours ranges from 17 to 54 months [1,11]. Despite different treatment strategies, gliomas are still characterized by a poor prognosis. A hallmark of most cancers is genomic instability , which refers to the accumulation of genomic alterations including point mutations, chromosomal aberrations, deletions, and amplifications during the cell cycle. This instability can manifest as microsatellite instability , a phenomenon in which short, repetitive DNA sequences (microsatellites) become highly variable due to defects in the DNA mismatch repair system (MMR), as well as chromosomal instability , characterized by an increased rate of chromosomal alterations, including both structural and numerical abnormalities [13,14]. Genomic instability has a dual role: it can cause catastrophic errors that threaten cell survival, but it also increases tumour heterogeneity, allowing the selection of more aggressive clones [15]. There is a limit to how much genomic damage a tumour cell can tolerate. When more than 75% of the tumour genome is damaged, the tumour often has a more favourable prognosis, probably because it has crossed a threshold of tolerance [15,16]. Genomic instability allows the accumulation of different genetic changes that leads to the formation of multiple genetically distinct clones within the same tumour, thereby creating tumour heterogeneity [15,17]. This heterogeneity provides the basis for clonal evolution, where different tumour cells compete, and a selective advantage is gained by those with traits that increase proliferation, invasiveness, or resistance to therapy [18,19]. As a result, over time, more aggressive clones dominate, leading to tumours with rapid growth, invasion, and therapy resistance. Gliomas are highly heterogeneous tumours, both morphologically and molecularly [20], enabled by genomic instability [21] which drives tumour promotion, progression and aggressiveness in gliomas [22,23]. Therefore, the aim of this study was to detect and measure the level of genomic instability in a larger sample of patients with astrocytoma and glioblastoma brain tumours. We suggest a potential role of genomic instability in the promotion and progression of these malignant brain tumours and the eventual development of new therapeutic protocols.

## 2. Results

### 2.1. Tissue Samples

Our 85 patients had brain tumours that were surgically removed and histopathologically and molecularly identified according to the classification issued by the WHO in 2021 [3]. All tumours belonged to the group of diffuse brain tumours, consisting of 57 glioblastoma IDH wild-type cases, and 28 astrocytoma IDH mutant cases. Blood samples from patients were collected alongside matched tumour tissue and served as normal control tissue for comparison.

### 2.2. Analysis of Genomic Instability in Malignant Brain Tumours

We analysed genomic instability in 85 patients with malignant brain tumours and corresponding control samples (blood of corresponding patients). For this purpose we calculated the frequency of total qualitative (MIN) and quantitative (CIN) DNA changes by dividing the total number of altered bands in the AP-PCR profile of tumour tissue by the total number of bands in the control tissue of the same patient. MIN and CIN equally contributed to the genomic instability of brain tumours in our cohort; that is, there were no differences in the frequency of changes between MIN and CIN (*p* = 0.44). Namely, the frequency of qualitative DNA changes was 0.176, the frequency of quantitative DNA changes was 0.172, and the frequency of total DNA changes was 0.348. Based on the frequency distribution of DNA alterations, we determined a cut-off value for each type of GI and each type of tumour, and accordingly divided the samples into two groups, one with high and the other with low GI (Table 1). The type and degree of genomic instability are significantly associated (*p* < 0.001), with low MIN being characteristic for our cohort. Fisher’s exact test revealed a statistically significant association between MIN and CIN in grade 3 tumours (*p* = 0.041); specifically, a decrease in MIN was accompanied by an increase in CIN.

**Figure 1:**
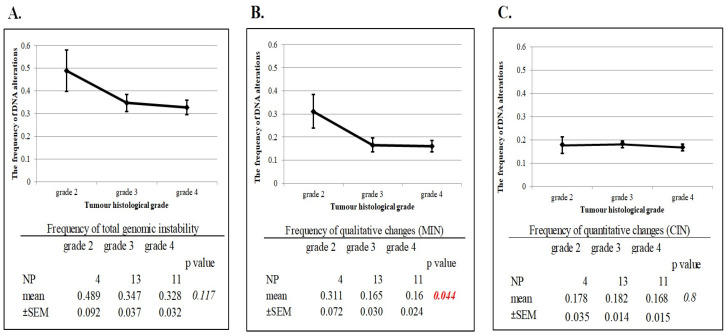
Genomic instability in astrocytoma of different grades. (**A**) Graphic representation of the relationship between the frequency of total genomic instability and the histological grades of astrocytoma. (**B**) Graphic representation of the relationship between the frequency of qualitative changes and the histological grades of astrocytoma (MIN). (**C**) Graphic representation of the relationship between the frequency of quantitative changes (CIN) and the histological grades of astrocytoma IDH mutant. NP—number of patients. The red-marked number indicates statistically significant values.

Further, we analysed the relationship between the frequency of DNA alterations and various clinicopathological parameters (Table 2). Using Fisher’s exact test, it was shown that there was an association between age and tumour type (*p* < 0.001). Patients older than 50 years often had glioblastoma, compared to younger patients who had astrocytoma. The types of brain tumour, the level of genomic instability and the age of patients are not independent events. Low GI was characteristic of older patients suffering from glioblastoma, while low GI was characteristic of patients in both age groups suffering from astrocytoma (*p* < 0.001). A low degree of CIN was characteristic for older patients with glioblastoma, while a high level of CIN was characteristic for younger patients with astrocytoma, and low CIN for older patients (*p* < 0.001). A low degree of MIN was characteristic of older patients with glioblastoma and younger patients with astrocytoma (*p* < 0.001). In examining the relationship between the frequency of DNA alterations and other clinicopathological parameters (gender, smoking status, tumour type, and histological grade), no statistically significant correlations were observed, except for the frequency of MIN among histological grades of astrocytoma (*p* = 0.044). Post hoc analysis revealed that grade 2 tumours exhibited a significantly higher frequency of qualitative changes compared to grade 3 and grade 4 tumours (*p* = 0.020 and *p* = 0.019, respectively), while the difference between grades 3 and 4 was not statistically significant. Also, we noted certain trends. Notably, even in the absence of statistical significance, a consistent and biologically meaningful trend was observed, indicating a decrease in frequency of total genomic instability with increasing tumour grade in astrocytoma (Figure 1).

Differences in the frequency of total genomic instability in relation to clinicopathological parameters are presented in Table 3. Among female patients, a statistically significant difference was observed between different grades of astrocytoma (*p* = 0.008). Grade 2 tumours exhibited a significantly higher level of total genomic instability compared to grades 3 and 4 (*p* = 0.004 and *p* = 0.005, respectively), while no significant difference was found between grades 3 and 4 (*p* = 0.669). A statistically significant difference was also observed between astrocytoma grades in non-smokers (*p* = 0.003), indicating that total genomic instability decreases with increasing tumour grade. Post hoc analysis revealed that grade 2 tumours had a significantly higher frequency of total genomic instability compared to grade 4 (*p* = 0.028), whereas no significant differences were found between grades 2 and 3 (*p* = 0.89) or between grades 3 and 4 (*p* = 0.067). The ANOVA (Analysis of Variance) test further demonstrated a significant difference in the frequency of total genomic instability between non-smokers, smokers, and former smokers within the group of patients with grade 3 tumours (*p* = 0.013). Post hoc analysis indicated that non-smokers had significantly higher levels of total genomic instability compared to smokers (*p* = 0.017) and former smokers (*p* = 0.013), while no significant difference was observed between smokers and former smokers (*p* = 0.891).

Among patients with astrocytoma grades 3 and 4, the ANOVA test revealed significant differences in the frequency of qualitative changes between smokers, non-smokers, and former smokers (*p* = 0.005; *p* = 0.016). Post hoc analysis using Fisher’s LSD method showed that smokers with grade 4 tumours had a significantly higher frequency of qualitative changes compared to non-smokers of the same grade (*p* = 0.016). Although differences were also observed between smokers and former smokers, as well as between non-smokers and former smokers, these did not reach statistical significance (*p* = 0.136 and *p* = 0.481, respectively). In non-smoking patients, a statistically significant difference in the frequency of qualitative changes was found across tumour grades 2, 3, and 4 (*p* = 0.006). Post hoc analysis revealed that grade 4 tumours had a significantly lower frequency of qualitative changes compared to grade 2 tumours (*p* = 0.033), whereas no significant differences were observed between grades 4 and 3 (*p* = 0.096) or between grades 3 and 2 (*p* = 0.832). A statistically significant difference was also observed between genders in grade 2, where female patients exhibited a significantly higher frequency of qualitative changes compared to male patients (*p* = 0.049). Furthermore, a statistically significant association was identified among female patients with astrocytoma across different tumour grades (*p* = 0.001). Post hoc analysis revealed significant differences between grades 2 and 3 (*p* < 0.001) and between grades 2 and 4 (*p* = 0.001), whereas no statistically significant difference was observed between grades 3 and 4 (*p* = 0.493) (Table 4).

The association between the frequency of quantitative changes and clinicopathological parameters is presented in Table 5. The ANOVA test revealed a significant association between tumour grade and gender in relation to the frequency of quantitative changes. Female patients with grade 4 tumours exhibited a high level of quantitative changes comparable to that observed in male patients (*p* = 0.019). Among male patients, statistically significant differences in the frequency of quantitative changes were observed between tumour grades (*p* = 0.032). In patients with astrocytoma, a significant difference in the level of quantitative changes was observed between non-smokers, smokers, and former smokers (*p* = 0.009). Post hoc analysis indicated that non-smokers had a significantly higher level of quantitative changes compared to smokers (*p* = 0.004), while no significant differences were found between non-smokers and former smokers (*p* = 0.076), or between smokers and former smokers (*p* = 0.741). A statistically significant association was also found between smoking status (non-smoker, smoker, and former smoker) and the frequency of quantitative changes in patients with astrocytoma grade 3 (*p* = 0.005). Among non-smokers with astrocytoma, no statistically significant association was observed across tumour grades (*p* = 0.053).

### 2.3. Analysis of Survival in Patients

**Figure 2:**
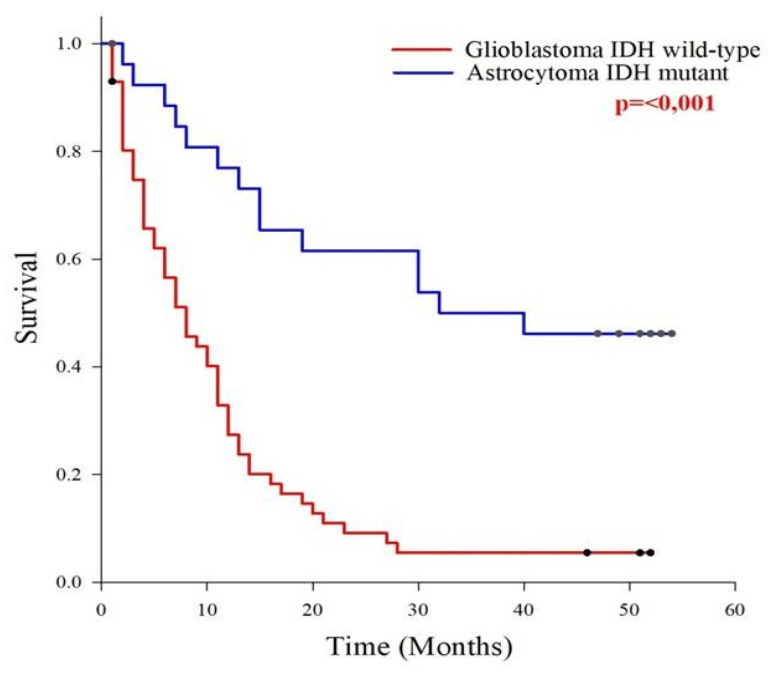
Survival analysis of types of brain tumours. Glioblastoma IDH wild-type OS 11.2, MST 8, astrocytoma IDH mutant OS 33.8, MST 32 months. OS, overall survival; MST, median survival time. Red-marked number indicates statistically significant value.

In our cohort the survival time was 18.5 months. A total of 25% of patients survived no more than 4 months, 50% survived no more than 11 months, and 75% did not survive beyond 27 months. The one-year survival rate was 41.2%, while the two-year survival rate was 24.7%. Using Kaplan–Meier (log-rank test) survival analysis, we examined patient survival in relation to the type and level of genomic instability, tumour type and grade, and various clinical–pathological parameters. For all three types of genomic instability, patients were divided into groups with high and low genomic instability. However, no statistically significant differences in survival were observed, indicating that the degree of genomic instability does not affect patient survival. Patients with glioblastoma IDH wild-type had significantly shorter survival than those with astrocytoma IDH mutant (*p* < 0.001). The average survival time for patients with glioblastoma IDH wild-type from surgery to death was 11.1 months, while patients with astrocytoma IDH mutant had an average survival time of 33.8 months (Figure 2).

**Figure 3:**
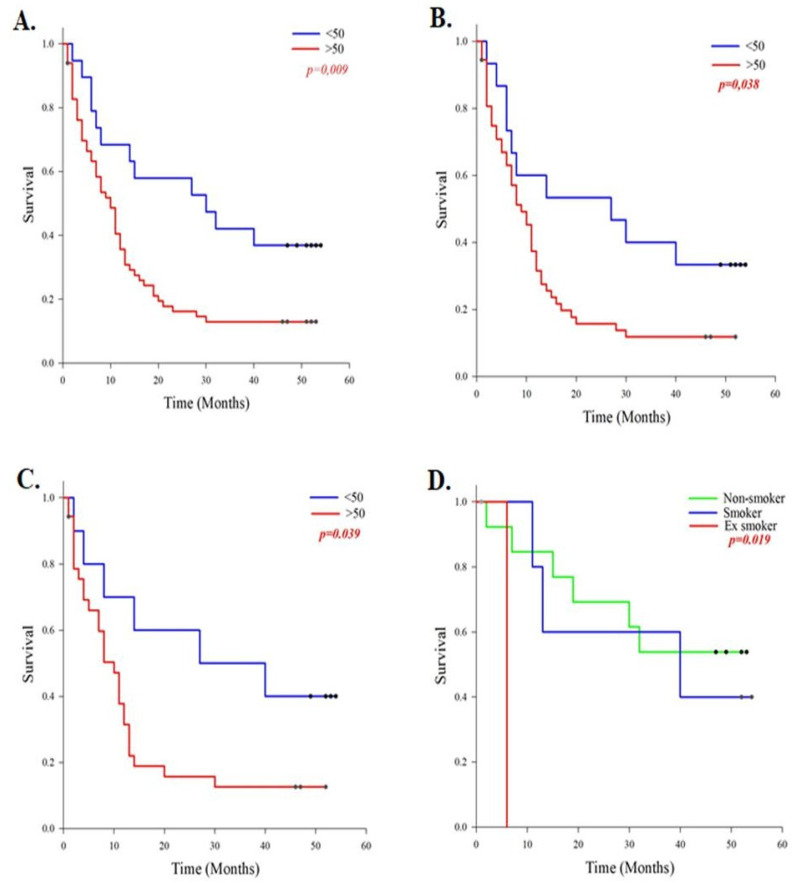
Survival of patients with malignant brain tumours in relation to clinicopathological parameters, the degree of genomic instability, and tumour type. (**A**) Survival of patients with malignant brain tumours according to age group: <50 OS 29.9, MST 30; >50 OS 14.8, MST 10. (**B**) Frequency of low qualitative changes in younger and older patients: <50 OS 27.6, MST 27; >50 OS 13.9, MST 9. (**C**) Frequency of low quantitative changes between younger and older patients: <50 OS 31.1, MST 27; >50 OS 13.9, MST 10. (**D**) Survival analysis of smoking status in astrocytoma IDH mutant patients: non-smoker OS 36.6, smoker OS 34.4, MST 40, former (ex) smoker OS 6, MST 6 months. OS, overall survival; MST, median survival time; red-marked numbers indicate statistically significant values.

Patients older than 50 years exhibited significantly poorer survival compared to younger patients (*p* = 0.009; Figure 3A). The median survival time for patients over 50 years of age was 14.8 months, whereas for those under 50 it was 29.9 months. Next, we analysed the impact of genomic instability on survival within these age groups. High or low overall genomic instability, as well as the degree of quantitative and qualitative alterations at a high frequency, did not significantly influence patient survival. However, both qualitative and quantitative changes occurring at a low frequency in patients older than 50 years were associated with poorer survival (*p* = 0.038 and *p* = 0.039, respectively; Figure 3B,C). Our study did not show that patient survival in malignant brain tumours is significantly associated with gender. Furthermore, no correlation was observed between the degree of genomic instability and malignant tumours in gender or between genders. When analysing individual tumour types, no significant association was identified between clinicopathological parameters and the frequency of genomic alterations in glioblastoma IDH wild-type or astrocytoma IDH mutant, except for smoking status, where former smokers exhibited poorer survival compared to current and non-smokers (*p* = 0.019; Figure 3D).

## 3. Discussion

We assessed genomic instability in 85 patients with malignant brain tumours using AP-PCR as a DNA profiling method. By comparing DNA profiles from tumour and normal tissue (blood of the same patient), we identified a significant degree of genomic instability in all cases. Instability was evident through both quantitative and qualitative alterations, reflecting the consequences of chromosomal and microsatellite instability. By comparing the mean values, we found that MIN and CIN contribute equally to the overall genomic instability in this cohort. A similar observation was previously reported by Milinkovic et al. (2012) and Martinez et al. (2004) [22,24]. CIN is more frequently observed and widely recognized as a main tumour driving factor of heterogeneity, disease progression, aggressiveness, and poor prognosis in malignant brain tumours [23,25,26]. However, our results clearly indicate that the dominant form of instability was low-degree microsatellite instability. This finding indicates the presence of subtle alterations in microsatellite sequences, reflecting a partially impaired but functionally active MMR system. Our results are consistent with previous studies reported by Tepeoglu et al. 2019 [27] and Rodríguez-Hernández et al. 2013 [28], who showed that a high level of MIN is a rare phenomenon in high-grade gliomas of adult patients. Type and degree of genomic instability have extremely important biological and therapeutic significance, as they provide insight into the fundamental mechanisms underlying tumour evolution and therapeutic resistance. Knowing the type of genomic instability is crucial for the choice of therapy, because certain chemotherapeutics can additionally stimulate MIN or CIN [29]. Despite the multidisciplinary treatment that is currently the standard of care [30], malignant brain tumours are still characterized by pronounced aggressiveness, rapid progression, and low overall patient survival rates. Low genomic instability in malignant brain tumours may contribute to excessive malignancy and increased aggressiveness, which was shown through survival analysis of our cohort. Although statistical significance was not demonstrated, low MIN, CIN and total genomic instability confer worse survival compared to high levels of instability in our cohort. Also, in brain tumours, low genomic instability could have implications for tumour evolution. In our study, we observed a correlation between the level of genomic instability and the histological grade of astrocytoma, with a trend indicating a decrease in genomic instability as tumour grade increases. These findings align with previous research that reported a similar pattern in lung cancers [31]. This suggests that high genomic instability during the early stages of tumorigenesis could play a key role in somatic evolution, promoting the emergence and selection of clones with enhanced survival potential, aggressiveness, and resistance to therapy. Genomic instability contributes to cancer progression by promoting intratumoral heterogeneity and generating diverse clones, some of which harbour mutations that confer adaptive advantages [19,32,33]. This process aligns with Darwinian evolutionary principles, where heterogeneous clones undergo natural selection and those carrying advantageous mutations are preferentially sustained [34,35]. According to our results, key selection occurs at grade 3, where we observed a decrease in MIN. However, CIN undergoes selective pressure only at grade 4, when its frequency also declines. This clearly shows a dynamic process of selection during tumour progression. This observation highlights tumour heterogeneity, wherein different clones with distinct genetic characteristics are selected at various stages of tumour development, influencing tumor aggressiveness and prognosis. In our cohort, MIN plays a statistically significant role, decreasing with higher tumour grade. A decrease in MIN as the grade increases could give advantage to tumour cells, as high levels of genomic instability could be associated with a more favourable prognosis [16,32,36,37,38], as such tumours are usually more susceptible to natural selection. In the absence of statistical significance, survival analysis showed that in our cohort, as grade increases, survival decreases; that is, the worst survival is at grade 4, with a survival time of 22.8 months, then grade 3, then grade 2 (survival times: 40.9 and 42.2 months, respectively). Glioblastoma arises as a primary, de novo tumour and does not undergo the same gradual, cross-selective evolutionary process, but is inherently very aggressive [39]. Our results show that glioblastoma has low levels of MIN, CIN, and overall GI, indicating the maintenance of genomic instability favourable for tumour survival but unfavourable for patient survival. In our cohort, median survival was only 8 months. Further, our results show that astrocytoma is common in younger patients, while glioblastoma predominates in older individuals, which is consistent with known epidemiology [6,40]. Also, patients older than 50 years demonstrated poorer survival compared to younger individuals, which can be associated with ageing [2,41,42] or different molecular and genetic factors. Our results indicate that low genomic instability may be associated with poor survival, specifically in patients older than 50 years with low CIN and low MIN. Low MIN, CIN and total instability occur more frequently in older patients with glioblastoma. As DNA damage accumulates with ageing, telomeres progressively shorten, and DNA repair efficiency declines [43,44], we would expect our patients to exhibit increased levels of genomic instability. Generally, glioblastoma is characterized by pronounced CIN [45,46]. Low CIN in glioblastoma, predominant in our samples, represents a stabilized genomic state compatible with tumour survival that could explain the discrepancy between our findings and published data. Astrocytoma exhibits low levels of MIN, CIN, and overall GI across both age groups in our cohort. However, in the literature, increased tumour aggressiveness and poorer clinical prognosis are associated with increased CIN in astrocytoma [25,26,47,48,49,50]. In our study, the level of CIN in astrocytoma may be low because excessive chromosomal instability threatens cell survival, so tumour cells maintain an optimal level of genomic instability during evolution, increasing their survival and viability. The literature also suggests that in elderly patients, MIN is mainly associated with sporadic epigenetic and genetic DNA damage and reduced repair [42,43], while in younger patients, MIN is often linked to hereditary cancer syndromes [51]. Recent studies suggest that high MIN in brain tumours may be associated with younger age through hereditary cancers [47,52]; our observation of consistently low MIN in astrocytoma across both age groups suggests that patients in our cohort are unlikely to carry hereditary genetic defects. In our analysis, low levels of MIN in both age groups in astrocytoma is consistent with the findings of Eckert et al. (2007) [53], whose results suggest that the incidence of MIN in paediatric and adult gliomas is extremely low. Gender is an important biological factor influencing cancer incidence, progression, survival, and treatment response. Glioblastoma is 1.6 times more frequent in men than in women, whereas astrocytoma is more commonly observed in women [54]. This underscores the importance of incorporating gender as a biological variable in the design and personalization of brain cancer treatment strategies. In our cohort, women with astrocytoma grade 2 showed significantly higher levels of MIN than men, whereas in grade 4 tumours women exhibited more pronounced CIN. Although MIN is generally uncommon in glial tumours [27,53], evidence from the literature indicates it occurs more frequently in women [27,55], as our results also show. CIN, which plays a central role in early gliomagenesis and is typically more prevalent in men [56,57], becomes more prominent in women at grade 4, and this could be the reason for the worse survival of women with this tumour grade, but the result is not statistically significant. These patterns suggest that the dynamics of genomic instability may be gender-specific. In female patients, the lower-grade tumours, in which MIN predominates, appear to gradually shift toward a chromosomal instability phenotype as they progress. We further analysed the relationship between smoking status and different types of genomic instability in malignant brain tumours. In non-smokers, tumour progression to higher grades was associated with a decrease in total GI and MIN. Interestingly, non-smokers with grade 3 astrocytoma exhibited a statistically significant increase in total GI and MIN compared to smokers and former smokers. Additionally, in astrocytoma, non-smokers showed a higher frequency of CIN relative to smokers and former smokers. However, the interpretation of these findings requires caution, as the results do not have sufficient statistical importance to draw definitive conclusions. The examined groups were small, particularly the cohorts of smokers and former smokers, and their composition was relatively homogeneous, which may limit the statistical importance and generalizability of the results. Comparison of survival outcomes in patients with astrocytoma revealed that former smokers, followed by current smokers, exhibited poorer survival compared to non-smokers. These observations suggest that smoking may exert a long-lasting influence on the behaviour of tumours, which persists even after cessation of tobacco exposure. Our results are consistent with the existing literature, which indicates that smoking contributes to the development of glial tumours, may promote an increased mutational burden and is associated with an increased risk of mortality [58,59,60,61].

## 4. Materials and Methods

### 4.1. Patients

Under the ethical standards of the Declaration of Helsinki from 1964, we received permission from the Ethics Committee of the University Clinical Center of Serbia and informed consent from 85 patients diagnosed with astrocytoma and glioblastoma who were operated on at the Clinic for Neurosurgery of the Clinical Center of Serbia. This enabled us to collect tumour and control tissue (blood) for further analysis. The tissue samples were frozen in liquid nitrogen immediately after the surgical intervention, and the control tissue was stored at 20 °C until the next steps in the analysis. Along with the samples, we received accompanying documentation that contained patient data, including gender, age, smoking status, survival data and tumour classification according to the criteria issued by the WHO in 2021 [3], in relation to tumour subtype with grade and diagnosed markers. In our sample, we had 57 patients diagnosed with glioblastoma IDH wild-type and 28 patients with astrocytoma IDH mutant, and 4 patients had astrocytoma IDH mutant grade 2, 13 patients had astrocytoma IDH mutant grade 3, and 11 patients had astrocytoma IDH mutant grade 4. The 85 patients included 42 men and 43 women, with a median age of 59.2 years (range: 15–79 years). The median survival time was 11 months. The smoking status was as follows: non-smokers, 43 patients; current smokers, 13 patients; and former smokers, 8 patients.

### 4.2. Isolation of Genomic DNA

For the isolation of genomic DNA, we used the phenol/chloroform/isoamyl alcohol method, a method according to Sambrook [62] with minor modifications. The concentration and purity of the genomic DNA were determined spectrophotometrically, and the quality was checked on a 0.8% agarose gel. 

### 4.3. Arbitrarily Primed PCR 

For the detection of genomic instability and the identification of genomic changes, we used the AP-PCR method, which is a DNA profiling method. With the AP-PCR method, we used random low-specificity primers in the first cycles of the PCR reaction, after which high-specificity conditions were applied to obtain DNA profiles with high numbers of bands. Optimization of AP-PCR reaction conditions was performed for each primer according to Cobb (1997) [63] and involved a search for conditions that would yield profiles of moderate complexity to simplify the analysis. Of the ten primers we tested, five primers (Mycrosynth, Balgach, CH) gave informative profiles: C-MYC 1 (Cellular Myelocytomatosis Oncogene), MDR 2 (Multi-Drug Resistance), BRAF 2 (B-Raf Proto-Oncogene, Serine/Threonine Kinase), CA12 F (Carbonic Anhydrase 12 forward) and CA9 R (Carbonic Anhydrase 9 reverse) (Table 6). AP-PCR DNA fingerprinting reactions for each primer were performed as previously described by Markovic et al. (2008) [31]. The PCR reaction products were separated by 6–8% non-denaturing polyacrylamide (PAA) gel, and for visualization, we used the silver staining method.

**Figure 4:**
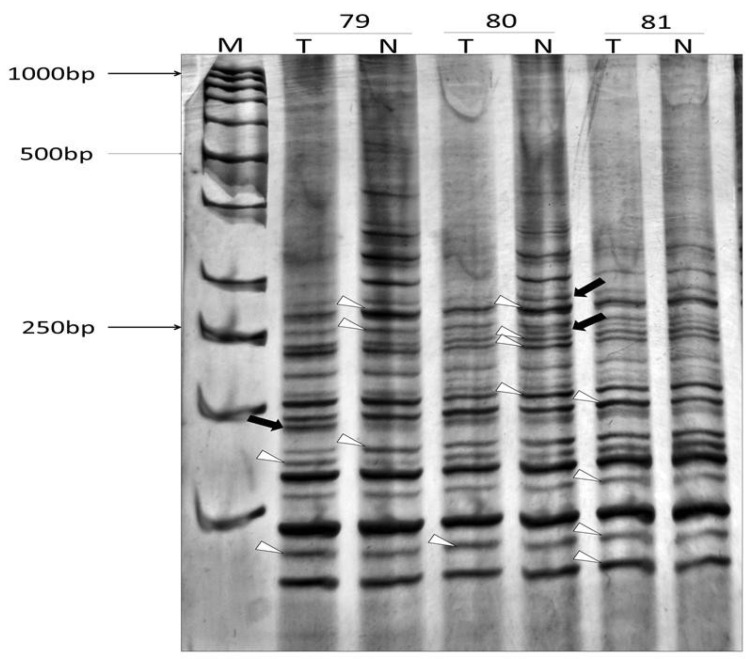
AP-PCR DNA profiles of tumour (T) and normal tissue (N) from patients with brain cancer, obtained with CA12 forward primer and separated at 6% non-denaturing PAA. Numbers 79–81 indicate patients; M indicates the DNA ladder; arrows indicate qualitative changes (MIN); arrowheads indicate quantitative changes (CIN).

An example of informative profiles of normal and tumour tissue from the same patient is shown in Figure 4.

**Figure 5:**
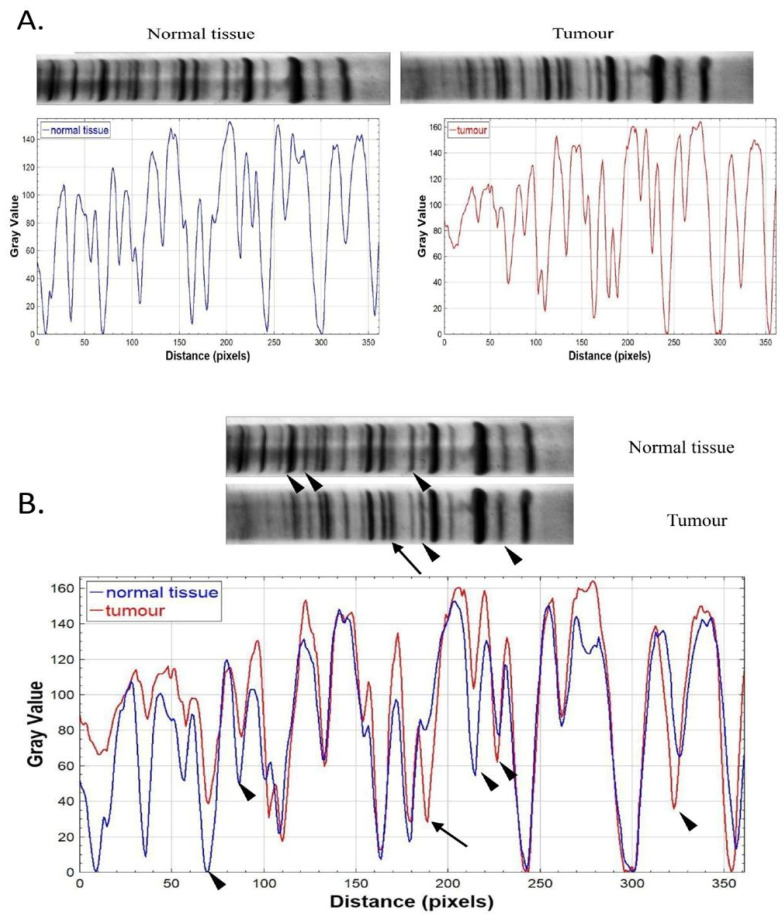
Analysis of genomic instability in a malignant brain tumour. (**A**) AP-PCR profiles and plot profiles of normal tissue and tumour tissues from the same patient. Edited using CLAHE method to improve local contrast with FIJI software. (**B**) By comparing the bottom of the profile between normal and tumour tissue, we observe qualitative changes (indicated by arrows) and quantitative changes (indicated by arrowheads), which are clearly visible when overlaying the plot profiles of tumour and normal tissue.

By comparing the DNA profiles of the tumour and control (blood) sample of the same patient, we observed two types of genetic differences, quantitative and qualitative, which were confirmed using FIJI 2.14.0 software (Madison, WI, USA) for image visualization using the method of adaptive histogram equalization with limited contrast (CLAHE) to improve local contrast (Figure 5A). Qualitative changes are observed as the presence or absence of bands and reflect microsatellite instability that occurs as a result of accumulated changes (insertions or deletions) in the DNA sequence. Quantitative changes are observed as differences in the intensity of bands that occur as a result of amplifications or deletions in randomly amplified regions and are an indicator of chromosomal instability (Figure 5B).

### 4.4. Statistical Analysis

We employed SigmaPlot 14.0 software (San Jose, CA, USA) for statistical analysis. The correlations between frequency alterations in genomic instability and clinical–pathological parameters were evaluated using Fisher’s exact test and the X^2^ test. To compare the mean values of samples, we used Student’s *t*-test for two groups or one-way ANOVA for comparing multiple groups for parametric data, while for non-parametric data, we used the Mann–Whitney test for two groups of data or Kruskal–Wallis for comparing multiple groups. The Kaplan–Meier test was utilized for survival analysis. Survival time was calculated from the day of surgery to the patient’s death or the last follow-up examination. Statistical significance was accepted for *p* ≤ 0.05.

## 5. Conclusions

Our study underlines the significant biological and clinical relevance of genomic instability, establishing it as an important driver of tumour evolution and clinical outcomes in malignant brain tumours. In our cohort, low genomic instability predominated, primarily manifesting as low-grade MIN. These findings highlight the critical importance of the correct characterization of the type and degree of genomic instability to enable a reliable modality for therapeutic treatment. In astrocytoma, the observed decrease in genomic instability with increasing tumour grade may reflect an adaptive stabilization of the genome, whereas glioblastomas maintain a balanced genomic homeostasis that facilitates aggressive tumour growth. These findings suggest that lower genomic instability may be associated with heightened malignancy and highlight the potential utility of assessing genomic instability as a prognostic marker for monitoring disease progression. These findings support our hypothesis that genomic instability contributes to tumour initiation, progression, and aggressiveness, highlighting its potential as a prognostic biomarker and a factor that can guide the selection of optimal therapeutic strategies in malignant brain tumours. We think that genomic instability has the potential to emerge as a critical prognostic biomarker and a promising target for the development of novel therapeutic strategies.

## Figures and Tables

**Table 1:** The levels of GI in analysed malignant brain tumours.

			Total Genomic Instability (GI)	Qualitative Changes (MIN)	Quantitative Changes (CIN)
Range			0.12–0.65	0.04–0.48	0.06–0.29
Cut-off			0.42	0.25	0.17
NP		High (%)	2 (25)	16 (19)	40 (47)
		Low (%)	64 (75)	69 (81)	45 (53)
*p*-value				<0.001	
Range		GBM	0.12–0.6	0.04–0.35	0.06–0.27
		AST	0.18–0.65	0.09–0.29	0.05–0.48
Cut-off		BGM	0.42	0.16	0.17
		AST	0.38	0.23	0.17
NP					
GBM	57	High (%)	13 (23)	26 (46)	27 (47)
		Low (%)	44 (77)	31 (54)	30 (53)
AST	28	High (%)	10 (36)	7 (25)	13 (46)
		Low (%)	18 (64)	21 (75)	15 (54)
*p*-value			*0.299*	*0.379*	*1.00*
Grade 4	11	High (%)	2 (18)	2 (18)	3 (27)
		Low (%)	9 (82)	9 (82)	8 (73)
*p*-value				1.00	
Grade 3	13	High (%)	2 (15)	2 (15)	8 (62)
		Low (%)	11 (85)	11 (85)	5 (38)
*p*-value				0.041	
Grade 2	4	High (%)	3 (75)	3 (75)	2 (50)
		Low (%)	1 (25)	1 (25)	2 (50)
*p*-value				1.00	

NP—number of patients; GBM—glioblastoma; AST—astrocytoma; red-marked numbers indicate statistically significant values.

**Table 2:** Association of patients with glioblastoma and astrocytoma according to age and level of genomic instability.

		Type of Brain Tumour				
		Glioblastoma		Astrocytoma		
Ages		<50	>50	<50	>50	*p*-Value
Number of patients		4	53	15	13	<0.001
	high	1	12	6	4	<0.001
GI	low	3	41	9	9	
	high	1	25	4	3	<0.001
13c MIN	low	3	28	11	10	
	high	1	26	8	5	<0.001
CIN	low	3	27	7	7	

Red-marked numbers indicate statistically significant values.

**Table 3:** Total genomic instability in types and grades of malignant brain tumours by clinicopathological parameters.

		Parameters									
		Gender			Age			Smoking Status			
		Female	Male	*p*-Value	<50	>50	*p*-Value	Non-Smoker	Smoker	Former Smoker	*p*-Value
GBM	NP	26	31		4	43		9	8	6	
	mean	0.353	0.335	0.551	0.29	0.347	0.413	0.335	0.323	0.384	0.564
	SEM	0.022	0.021		0.059	0.015		0.021	0.036	0.052	
AST	NP	17	11					14	5	2	
	mean	0.375	0.337	0.493	15	13	0.434	0.387	0.373	0.262	0.502
	SEM	0.034	0.041		0.381	0.335		0.035	0.077	0.067	
*p*-value		0.597	0.956		0.037	0.036		0.21	0.573	0.266	
Grade 4	NP	8	3		8	3		5	2	1	
	mean	0.158	0.165	0.341	0.322	0.342	0.63	0.276	0.395	0.329	0.395
	SEM	0.032	0.033		0.037	0.065		0.024	0.063		
Grade 3	NP	6	7		5	8		7	1	1	
	mean	0.129	0.197	0.45	0.407	0.31	0.299	0.419	0.231	0.195	0.013
	SEM	0.018	0.052		0.076	0.036		0.057			
Grade 2	NP	3	1		2	2		2	2		
	mean	0.373	0.125	0.129	0.555	0.423	0.637	0.555	0.427		0.637
	SEM	0.053			0.005	0.206		0.044	0.206		
*p*-value		0.008	0.247		0.221	0.904		0.003	0.533		0.767

NP—number of patients; GBM—glioblastoma; AST—astrocytoma; red-marked numbers indicate statistically significant values.

**Table 4:** Qualitative changes in type and grade of malignant brain tumours by clinicopathological parameters.

		Parameters									
		Gender			Age			Smoking Status			
		Female	Male	*p*-Value	<50	>50	*p*-Value	Non-Smoker	Smoker	Former Smoker	*p*-Value
GBM	NP	26	31		4	43		9	8	6	
	mean	0.185	0.162	0.152	0.133	0.175	0.186	0.163	0.147	0.202	0.397
	SEM	0.014	0.015		0.024	0.011		0.013	0.024	0.042	
AST	NP	17	11		15	13		14	5	2	
	mean	0.186	0.182	0.962	0.192	0.175	0.645	0.194	0.237	0.117	0.5
	SEM	0.028	0.034		0.029	0.032		0.030	0.071	0.029	
*p*-value		0.495	0.753		0.15	0.562		0.542	0.28	0.57	
Grade 4	NP	8	3		8	3		5	2	1	
	mean	0.158	0.165	0.882	0.149	0.190	0.565	0.106	0.252	0.146	0.016
	SEM	0.032	0.033		0.023	0.059		0.019	0.050		
Grade 3	NP	6	7		5	8		7	1	1	
	mean	0.129	0.197	0.25	0.209	0.138	0.364	0.221	0.082	0.087	0.005
	SEM	0.018	0.052		0.068	0.024		0.043			
Grade 2	NP	3	1		2	2		2	2		
	mean	0.373	0.125	0.049	0.321	0.301	0.927	0.321	0.301		0.927
	SEM	0.053			0.018	0.176		0.018	0.176		
*p*-value		0.001	0.555		0.143	0.448		0.006	0.33	0.667	

NP—number of patients; GBM—glioblastoma; AST—astrocytoma; red-marked numbers indicate statistically significant values.

**Table 5:** Quantitative changes in type and grades of malignant brain tumours by clinicopathological parameters.

		Parameters									
		Gender			Age			Smoking Status			
		Female	Male	*p*-Value	<50	>50	*p*-Value	Non-Smoker	Smoker	Former Smoker	*p*-Value
GBM	NP	26	31		4	53		29	8	6	
	mean	0.168	0.173	0.741	0.157	0.172	0.719	0.173	0.175	0.182	0.919
	SEM	0.010	0.009		0.037	0.007		0.010	0.015	0.017	
AST	NP	17	11		15	13		14	5	2	
	mean	0.189	0.155	0.075	0.190	0.160	0.121	0.193	0.136	0.146	0.009
	SEM	0.013	0.013		0.014	0.012		0.009	0.012	0.037	
*p*-value		0.213	0.277		0.455	0.413		0.132	0.066	0.499	
Grade 4	NP	8	3		8	3		5	2	1	
	mean	0.183	0.126	0.019	0.174	0.152	0.357	0.171	0.143	0.183	0.275
	SEM	0.017	0.010		0.020	0.009		0.015	0.014		
Grade 3	NP	6	7		5	8		7	1	1	
	mean	0.188	0.177	0.745	0.198	0.172	0.409	0.198	0.149	0.108	0.005
	SEM	0.028	0.013		0.023	0.018		0.009			
Grade 2	NP	3	1		2	2		2	2		
	mean	0.207	0.092	0.112	0.234	0.122	0.131	0.234	0.122		0.131
	SEM	0.028			0.012	0.030		0.012	0.030		
*p*-value		0.829	0.032		0.364	0.38		0.053	0.4	0.667	

NP—number of patients; GBM—glioblastoma; AST—astrocytoma; red-marked numbers indicate statistically significant values.

**Table 6:** Primer sequences and AP-PCR conditions.

Primer Name	Primer Sequence	AP-PCR Low-Stringency Conditions for the First 5 Cycles	AP-PCR High-Stringency Conditions for the Remaining 35 Cycles	AP-PCR Reaction Mixture
C-MYC 1	5′-GCT CCA AGA CGT TGT GTG TTC G-3′	94 °C 1’; 45 °C 2’; 72 °C 2’	94 °C 1’; 65 °C 1’; 72 °C 2’	2 mM MgCl_2_; 0.6 mM dNTP; 5.5 µM primer; 35 ng DNA
MDR 2	5′-GTT CAA ACT TCT GCT CCT GA-3′	95 °C 30 s; 45 °C 2’; 72 °C 1’	95 °C 30 s; 60 °C 1’; 72 °C 1’	2.5 mM MgCl_2_; 0.4 mM dNTP; 5 µM primer; 50 ng DNA
BRAF 2	5′-GAC CCC ACT CCA TCG AGA TTT-3′	95 °C 30 s; 40 °C 2’; 72 °C 1’	95 °C 30 s; 60 °C 1’; 72 °C 1’	2.5 mM MgCl_2_; 0.4 mM dNTP; 5 µM primer; 50 ng DNA
CA12 F	5′-ACT GCG GCA GGA CTG AGT CT-3′	95 °C 30 s; 40 °C 2’; 72 °C 1’	95 °C 30 s; 60 °C 1’; 72 °C 1’	1.5 mM MgCl_2_; 0.6 mM dNTP; 5 µM primer; 50 ng DNA
CA9 R	5′-CCT CCA TAG CGC CAA TGA CT-3′	95 °C 30 s; 40 °C 2’; 72 °C 1’	95 °C 30 s; 60 °C 1’; 72 °C 1’	1.5 mM MgCl_2_; 0.6 mM dNTP; 5 µM primer; 50 ng DNA

## Data Availability

The original contributions presented in this study are included in the article. Further inquiries can be directed to the corresponding author.

## Abbreviations

The following abbreviations are used in this manuscript:

IDH	Isocitrate Dehydrogenase
MIN, MSI	Microsatellite Instability
CIN	Chromosomal Instability
GI	Genomic Instability
AP-PCR	Arbitrarily Primed Polymerase Chain Reaction
WHO	World Health Organization
EGFR	Epidermal Growth Factor Receptor
PTEN	Phosphatase and TENsin Homolog
TERT	Telomerase Reverse Transcriptase
TP53	Tumour Protein 53
ATRX	Alpha-thalassemia Intellectual Disability, X-linked
MGMT	O6-Methylguanine-DNA Methyltransferase
CNS	Central Nervous System
DNA	Deoxyribonucleic Acid
PAA	Polyacrylamide
CLAHE	Contrast-limited Adaptive Histogram Equalization
ANOVA	Analysis of Variance
C-MYC 1	Cellular Myelocytomatosis Oncogene
MDR 2	Multi-Drug Resistance
BRAF 2	B-Raf Proto-Oncogene, Serine/Threonine Kinase
CA12 F	Carbonic Anhydrase 12 Forward
CA 9 R	Carbonic Anhydrase 9 Reverse
dNTP	Deoxyribonucleoside Triphosphate
MgCl2	Magnesium Chloride
GBM	Glioblastoma IDH Wild-type
AST	Astrocytoma IDH Mutant
OS	Overall Survival
MST	Median Survival Time
TMZ	Temozolomide
MMR	Mismatch Repair
SEM	Standard Error of the Mean
ICI	Immune Checkpoint Inhibitor
